# Regional pulse wave velocities and their cardiovascular risk factors among healthy middle-aged men: a cross-sectional population-based study

**DOI:** 10.1186/1471-2261-14-5

**Published:** 2014-01-13

**Authors:** Jina Choo, Chol Shin, Emma Barinas-Mitchell, Kamal Masaki, Bradley J Willcox, Todd B Seto, Hirotsugu Ueshima, Sunghee Lee, Katsuyuki Miura, Lakshmi Venkitachalam, Rachel H Mackey, Rhobert W Evans, Lewis H Kuller, Kim Sutton-Tyrrell, Akira Sekikawa

**Affiliations:** 1 College of Nursing, Korea University, Anam-Dong, Seongbuk-Gu, Seoul 136-705, South Korea; 2 Department of Epidemiology, Graduate School of Public Health, University of Pittsburgh, Pittsburgh, PA, USA; 3 Department of Health Science, Shiga University of Medical Science, Otsu, Japan; 4 Division of Pulmonary and Critical Care Medicine, Department of Internal Medicine, Korea University Ansan Hospital, Ansan, South Korea; 5 The Queen’s Medical Center, Honolulu, HI, USA; 6 Department of Gerontology, University of Hawaii, Honolulu, HI, USA; 7 Department of Biomedical and Health Informatics, School of Medicine, University of Missouri-Kansas City, Kansas, MO, USA

**Keywords:** Arterial stiffness, Aorta, Carotid arteries, Brachial artery, Femoral artery

## Abstract

**Background:**

Both carotid-femoral (*cf*) pulse wave velocity (PWV) and brachial-ankle (*ba)* PWV employ arterial sites that are not consistent with the path of blood flow. Few previous studies have reported the differential characteristics between *cf*PWV and *ba*PWV by simultaneously comparing these with measures of pure central (aorta) and peripheral (leg) arterial stiffness, i.e., heart-femoral (*hf)* PWV and femoral-ankle (*fa*) PWV in healthy populations. We aimed to identify the degree to which these commonly used measures of *cf*PWV and *ba*PWV correlate with *hf*PWV and *fa*PWV, respectively, and to evaluate whether both *cf*PWV and *ba*PWV are consistent with either *hf*PWV or *fa*PWV in their associations with cardiovascular (CV) risk factors.

**Methods:**

A population-based sample of healthy 784 men aged 40–49 (202 white Americans, 68 African Americans, 202 Japanese-Americans, and 282 Koreans) was examined in this cross-sectional study. Four regional PWVs were simultaneously measured by an automated tonometry/plethysmography system.

**Results:**

*cf*PWV correlated strongly with *hf*PWV (r = .81, *P* < .001), but weakly with *fa*PWV (r = .12, *P* = .001). *ba*PWV correlated moderately with both *hf*PWV (r = .47, *P* < .001) and *fa*PWV (r = .62, *P* < .001). After stepwise regression analyses with adjustments for race, *cf*PWV shared common significant correlates with both *hf*PWV and *fa*PWV: systolic blood pressure (BP) and body mass index (BMI). However, BMI was positively associated with *hf*PWV and *cf*PWV, and negatively associated with *fa*PWV. *ba*PWV shared common significant correlates with *hf*PWV: age and systolic BP. *ba*PWV also shared the following correlates with *fa*PWV: systolic BP, triglycerides, and current smoking.

**Conclusions:**

Among healthy men aged 40 – 49, *cf*PWV correlated strongly with central PWV, and *ba*PWV correlated with both central and peripheral PWVs. Of the CV risk factors, systolic BP was uniformly associated with all the regional PWVs. In the associations with factors other than systolic BP, *cf*PWV was consistent with central PWV, while *ba*PWV was consistent with both central and peripheral PWVs.

## Background

Increased arterial stiffness is a strong predictor of the risk for cardiovascular disease in patients with hypertension as well as in the general population independent of cardiovascular (CV) risk factors [1-4]. Arterial stiffness is mainly related to the aging process and high blood pressure and can be accelerated by CV risk factors [5-8].

Arterial stiffness can be noninvasively evaluated by measuring pulse-wave velocity (PWV). The PWV is calculated using distance and time: the path length between two arterial sites and the amount of time required for the pressure wave to travel from one arterial site to the next. Carotid-femoral PWV (*cf*PWV) measured by Doppler ultrasound is the most widely used measure of aortic stiffness and is recognized as the gold standard measure for evaluating arterial stiffness. Alternatively, brachial-ankle PWV (*ba*PWV) measured by the Omron oscillometric/plethysmographic system has recently received attention because of its consistently high association with CV risk factors and its ease of use for large-scale population studies [9-13]. Increased *ba*PWV has been reported to be an independent predictor of all-cause mortality in the general population [11], as has *cf*PWV [3].

Both *cf*PWV and *ba*PWV employ arterial sites that are not consistent with the path of blood flow. To adjust for this, an assumption is made about the timing of the pressure wave travelling the opposite direction and this is used to adjust the measure accordingly. For example, when measuring *cf*PWV, the blood does not flow from the carotid artery to the femoral artery. Thus, an assumption is made that the time that the pressure wave takes to travel from the heart to the carotid artery is the same amount of time that it takes a simultaneous wave to travel the same distance from the heart to the descending aorta. Thus, *cf*PWV, while using the carotid and femoral arterial sites, actually represents the velocity of the pressure wave from the descending aorta to the femoral artery.

Current technology allows us to simultaneously measure PWVs between multiple arterial sites. Based on the assumptions used to calculate the measures, *cf*PWV should reflect the stiffness of the descending aorta, while *ba*PWV should reflect the stiffness of both the descending aorta and the leg artery. To evaluate this, *cf*PWV and *ba*PWV can be compared with PWV measures that are more directly derived from contiguous points along the path of blood flow. We have chosen heart-femoral PWV (*hf*PWV) as a measure of central (aortic) stiffness and femoral-ankle PWV (*fa*PWV) as a measure of peripheral (leg) stiffness. The comparison between *ba*PWV and *cf*PWV as measures of arterial stiffness has been reported in a few studies [12,13]. However, there is no information on whether both *ba*PWV and *cf*PWV are comparable with either *hf*PWV or *fa*PWV in these measurements of arterial stiffness.

Differentiating central vs. peripheral PWV is important because they are different pathophysiologically. The central arteries become stiffer with aging because of a decreased ratio of elastin to collagen [14-16]. The peripheral arteries that contain a greater proportion of smooth muscle cells may not be similarly influenced by aging [7]. However, the degree to which CV risk factors affect central vs. peripheral arteries differently is not yet clear. A few studies have reported characteristics of *ba*PWV, *hf*PWV and *fa*PWV in their associations with CV risk factors in populations with hypertension or type II diabetes mellitus [17,18]. However, no previous studies have reported such differential characteristics between *cf*PWV vs. *ba*PWV by simultaneously comparing these with *hf*PWV or *fa*PWV in healthy population groups.

The purpose of this study was to identify the degree to which the commonly used measures of *cf*PWV and *ba*PWV correlated with measures of central and peripheral PWVs (i.e., *hf*PWV and *fa*PWV) which use the arterial sites that are contiguous along the path of blood flow. In addition, we evaluated whether both *cf*PWV and *ba*PWV were consistent with either *hf*PWV or *fa*PWV in their associations with CV risk factors. We conducted the present study in a healthy population sample of White, African, and Japanese-American as well as Korean men of the ERA-JUMP Study.

## Methods

### Study participants

During 2002 to 2006, a population-based sample of 1,022 men aged 40–49 was randomly selected by three centers of the ERA-JUMP Study: 310 White Americans and 107 African Americans from Allegheny County, PA, U.S.; 303 Japanese-Americans from Honolulu, HI, U.S.; 302 Koreans from Ansan, Gyeonggi-do, South Korea [19-22]. Originally, the ERA-JUMP study consisted of a population sample of the Japanese population whose PWV measures had not been examined. All participants were without clinical cardiovascular disease or other severe diseases [19]. For the purpose of recruiting healthy participants for the present study, we excluded those with peripheral arterial disease, hypertension, diabetes, or hyperlipidemia. Of the original samples, we excluded (1) those having ≤ 0.9 of either left or right ankle-brachial index (n = 7), (2) those taking antihypertensive, diabetes, or lipid-lowering medications (n = 166); and (3) those with values missing for all four regional PWVs (i.e., *hf*PWV, *cf*PWV, *fa*PWV, and *ba*PWV) (n = 62). The final sample was 784 (232 White Americans, 68 African Americans, 202 Japanese-Americans, and 282 Koreans).

Written informed consent was obtained from all participants. The study was approved by the Institutional Review Boards of the following institutions: the University of Pittsburgh, Pittsburgh, PA, U.S.; the Kuakini Medical Center, Honolulu, HI, U.S.; and Korea University, Seoul, South Korea.

### PWV measurement

The PWV was automatically generated using a noninvasive and automated waveform analyzer (Colin-VP2000/1000, Omron, Japan/WaveNexus,TX). This device records the electrocardiogram (ECG), phonocardiogram, and pressure waveforms. ECG electrodes were placed on both wrists. A phonocardiogram was placed on the left edge of the 4^th^-rib-case; heart sounds S_1_ and S_2_ were detected. Pressure waveforms of the brachial and tibial arteries were recorded by a plethysmographic sensor and an oscillometric pressure sensor using the occlusion/sensing cuffs adapted to both arms (i.e., brachial arteries) and both ankles (i.e., tibial arteries). Pressure waveforms of the carotid and femoral arteries were recorded using multiarray tonometry sensors placed at the left carotid and the left femoral arteries. Following 10 minutes of rest in a supine position, pressure waveforms were stored for a sampling time of 10 seconds with automatic gain analysis and quality adjustment.

PWV (cm/second) was calculated as (path length between arterial sites)/(time interval). The path length for *cf*PWV was obtained by using the actual distance of participants’ body (in cm): ([cm from suprasternal notch to bottom of umbilicus] + [cm from botton of umbilicus to femoral sensor]) – [cm from carotid to suprasternal notch]. Furthermore, the path length for the heart-brachial (D*hb*), the heart-femoral (D*hf*), the heart-ankle (D*ha*), the femoral-ankle (D*fa*), and the brachial-ankle (D*ha* – D*hb*) segments were obtained using the following formulas: D*hb =* 0.2195×height – 2.0734; D*hf* =0.5643×height – 18.381; D*ha* = 0.8129×height + 12.328; D*fa =* 0.2486×height – 30.709. The PWV results were obtained with the left sides of *cf*, *ba* and *fa* site*s*. Reproducibility of the automated PWV measurement was assessed by the University of Pittsburgh Ultrasound Research Laboratory, and used 19 and 12 participants for obtaining intraclass and interclass correlation coefficients, respectively. Intraclass correlation coefficients of 0.86 (*hf*PWV), 0.76 (*cf*PWV), 0.96 (*fa*PWV), and 0.97 (*ba*PWV) were obtained within technicians. Interclass correlation coefficients of 0.93 (*hf*PWV), 0.73 (*cf*PWV), 0.87 (*fa*PWV), 0.91 (*ba*PWV) were achieved between technicians [23].

We standardized the PWV measurement across the 3 centers. Before the study started, staff at the Ultrasound Research Laboratory, University of Pittsburgh visited Honolulu to train the sonographers in Honolulu and from South Korea in PWV measurements.

### Other measurements

All participants underwent a physical examination, and completed a lifestyle questionnaire (e.g., current smokers, alcohol drinking [two times or greater per week], and use of anti-hypertensives, diabetic medications, or lipid-lowering medications) and a laboratory assessment as described previously [20]. Venipuncture was performed early in the clinic visit after a 12-h fast. Samples were storied at -80°C and shipped on dry ice to the University of Pittsburgh [20]. Biochemical measurements of the blood samples were centralized at the Heinz Laboratory of the University of Pittsburgh. Serum lipids were determined with the standardized methods according to the Centers for Disease Control and Prevention, including total cholesterol, low-density-lipoprotein cholesterol (LDL-C), high-density-lipoprotein cholesterol (HDL-C), and triglycerides [24]. Serum fasting glucose was determined by a hexokinase -glucose-6-phosphate-dehydrogenase-enzymatic assay; serum fasting insulin by a radioimmunoassay (Linco Research Inc., Saint Charles, MO). Blood pressure (BP) (mmHg) and heart rate (beats per minute) were measured at right brachial artery in the sitting position after a 5-minute rest with the bladder emptied, by using an automated sphygmomanometer (BP-8800, Colin Medical Technology, Komaki, Japan) at the clinic visit. An average of two measurements was used. All the data collection was standardized across all 3 centers.

### Statistical analysis

We analyzed the pooled data of White Americans (n = 232), African Americans (n = 68), Japanese-Americans (n = 202), and Koreans (n = 282). Sociodemographic and clinical characteristics and regional PWVs were described by using means (standard deviations), medians (interquartile ranges), frequencies, and percentages as appropriately. To obtain correlation coefficients, we performed the partial correlation analysis with adjustment for age and race. To examine race-adjusted associations between PWVs and CV risk factors, we performed multiple regression analysis by placing each CV risk factor as a predictor variable in the model after adjusting for race (placed in dummy variables). Next, to identify CV risk factors associated with each PWV, we performed stepwise regression analysis with the backward selection that specified the significance level (*P* value > .1) with adjustment for race as a lockterm. The CV risk factors included as predictor variables into the models were age, systolic BP, heart rate, body mass index (BMI), current smoking status, LDL-C, the ratio of total cholesterol vs. HDL-C, triglycerides (log-transformed), fasting glucose, and fasting insulin (log-transformed). Statistical significance was considered to be *P* < .05. All statistical analyses were performed with STATA 10.0 for Windows (StataCorp LP, College Station, TX).

## Results

Participants (N = 784) had a mean age of 45.1years and a mean BMI of 26.4kg/m^2^. They had an average of BP of 122.9/74.7mmHg (Table 1). Of 784 subjects, 21.6% and 41.6% were current smokers and alcohol drinkers, respectively.

**Table 1:** Clinical characteristics of the study participants in 2002–2006 (N = 784)

	**n (%)**	**Mean (SD)**
Race		
White Americans	232 (29.5)	
African Americans	68 (8.7)	
Japanese Americans	202 (25.8)	
Koreans	282 (36.0)	
Age, years		45.1 (2.9)
Systolic BP, mmHg		122.9 (12.8)
Diastolic BP, mmHg		74.7 (9.9)
Heart rate, beats/min		65.7 (9.3)
BMI, kg/m^2^		26.4 (4.0)
Total cholesterol, mg/dL		205.6 (38.0)
LDL-C, mg/dL		126.8 (34.5)
HDL-C, mg/dL		48.4 (13.0)
Total cholesterol/HDL-C ratio		4.49 (1.23)
Triglycerides, mg/dL		129.0 (91.0 – 195.0)
Fasting glucose, mg/dL		103.0 (15.6)
Fasting insulin, μIU/dL		11.0 (8.4 – 15.0)
Current smoking	169 (21.6)	
Alcohol drinking	326 (41.6)	

Values are expressed as means (standard deviations) or median (interquartile range) for continuous variables and total number (percentages) for categorical variables.

*BMI* body mass index, *BP* blood pressure, *HDL-C* high density lipoprotein cholesterol, *LDL-C* low density lipoprotein cholesterol, *SD* standard deviation.

**Figure 1:**
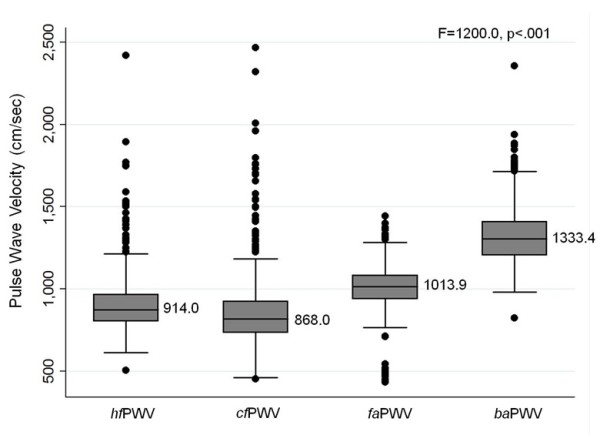
**Region-specific distribution of pulse wave velocity (N = 784).** PWV = pulse wave velocity, *hf* = heart-femoral, *cf* = carotid-femoral, *fa* = femoral-ankle, *ba* = brachial-ankle. Each line on a box indicates a median value of each regional PWV. F and P values indicate significant difference across all the regional PWVs.

The PWV values significantly differed by arterial regions (F = 1,200.0, *P* < .001) (Figure 1). *ba*PWV had the highest value, followed by *fa*PWV, *hf*PWV, and *cf*PWV (*P* < .001). The peripheral *fa*PWV was significantly higher than the central *hf*PWV (*P* < .001) and *cf*PWV (*P* < .001).

### Age-and race-adjusted correlations between PWVs

All the regional PWVs correlated significantly with each other. The correlations of *cf*PWV and *ba*PWV with either *hf*PWV or *fa*PWV were not independent (Table 2). *cf*PWV correlated strongly with *hf*PWV (r = .81, *P* < .001), but weakly with *fa*PWV (r = .12, *P* = .001). *ba*PWV correlated moderately with both *hf*PWV (r = .47, *P* < .001) and *fa*PWV (r = .62, *P* < .001). According to the data from this correlation matrix, *cf*PWV mostly reflected properties of central arterial stiffness, while *ba*PWV reflected mixed properties of both central and peripheral arterial stiffness. *ba*PWV moderately correlated with *cf*PWV (r = .42, *P* < .001). Meanwhile, the correlation between *hf*PWV and *fa*PWV was also significant, but minimal (r = .08, *P* = .030).

**Table 2:** **Correlations**^**a **^**between regional pulse wave velocity values (N = 784)**

**Regional PWV**	**r (*P *** **value)**			
	***hf*** **PWV**	***cf*** **PWV**	***fa*** **PWV**	***ba*** **PWV**
*hf*PWV	1.00			
*cf*PWV	0.81 (<.001)	1.00		
*fa*PWV	0.08 (.030)	0.12 (.001)	1.00	
*ba*PWV	0.47 (<.001)	0.42 (<.001)	0.62 (<.001)	1.00

^a^Partial correlation was performed with adjustment for age and race. PWV pulse wave velocity, *hf* heart-femoral, *cf* carotid-femoral, *fa* femoral-ankle, *ba* brachial-ankle.

### Race-adjusted associations of CV risk factors with regional PWVs

Age was significantly and positively associated with *hf*PWV and *ba*PWV (Table 3). Systolic BP and heart rate were significantly and positively associated with all the regional PWVs. Similarly, triglycerides and fasting insulin were significantly and positively associated with all the regional PWVs; however, LDL-C was not significantly associated with any regional PWV.

**Table 3:** **Associations**^**a **^**between regional pulse wave velocity and cardiovascular risk factors (N = 784)**

	**Beta (*P *** **value)**							
	***hf*** **PWV**		***cf*** **PWV**		***fa*** **PWV**		***ba*** **PWV**	
Age, years	.15	(<.001)	.06	(.074)	.05	(.187)	.09	(.012)
Systolic BP, mmHg	.39	(<.001)	.31	(<.001)	.33	(<.001)	.47	(<.001)
Heart rate, beats/min	.09	(.016)	.11	(.002)	.18	(<.001)	.21	(<.001)
BMI, kg/m^2^	.23	(<.001)	.31	(<.001)	.02	(.526)	.18	(<.001)
LDL-C, mg/dL	.06	(.121)	-.01	(.697)	-.01	(.721)	.01	(.692)
Total cholesterol/HDL-C ratio	.11	(.003)	.12	(.001)	.05	(.131)	.12	(<.001)
Triglycerides, mg/dL	.16	(<.001)	.18	(<.001)	.20	(<.001)	.27	(<.001)
Fasting glucose, mg/dL	.06	(.111)	.06	(.080)	.08	(.020)	.10	(.003)
Fastinginsulin, μIU/dL	.15	(<.001)	.16	(<.001)	.08	(.026)	.16	(<.001)
Current smoking, (%)	.03	(.398)	.06	(.116)	.12	(.002)	.09	(.012)
Alcohol drinking, (%)	-.00	(.983)	-.04	(.308)	.10	(.005)	.06	(.075)

^a^Resulted from the multiple regression analysis after adjustment for race. *BMI* body mass index, *BP* blood pressure, *HDL-C* high density lipoprotein cholesterol, *LDL-C* low density lipoprotein cholesterol, *PWV* pulse wave velocity, *hf* heart-femoral, *cf* carotid-femoral, *fa* femoral-ankle, *ba* brachial-ankle.

BMI was significantly and positively associated with *hf*PWV, *cf*PWV, and *ba*PWV, but not with *fa*PWV. Current smoking was significantly and positively associated with *fa*PWV and *ba*PWV.

### Multivariable adjusted associations of CV risk factors with regional PWVs

After the stepwise regression analyses with adjustments for race, the CV risk factors of age, systolic BP, BMI, heart rate, triglycerides, and current smoking were included in the regression models as significant correlates for at least one regional PWV (Table 4). Significant correlates for *hf*PWV were age, systolic BP, and BMI. Significant correlates for *cf*PWV were systolic BP and BMI. Significant correlates for *fa*PWV were systolic BP, BMI, triglycerides, and current smoking. Significant correlates for *ba*PWV were age, systolic BP, heart rate, triglycerides, and current smoking. As a result, all the regional PWVs had a common significant correlate, i.e., systolic BP. In particular, *cf*PWV shared common correlates with both *hf*PWV and *fa*PWV (i.e., systolic BP and BMI). However, BMI was significantly and positively associated with *hf*PWV and *cf*PWV, and negatively associated with *fa*PWV. Meanwhile, *ba*PWV shared common correlates with *hf*PWV (i.e., age and systolic BP) and also with *fa*PWV (i.e., systolic BP, triglycerides, and current smoking).

**Table 4:** **Stepwise regression analysis**^**a**^**: Associations between pulse wave velocity and cardiovascular risk factors (N = 784)**

	**beta**	***P *** **value**	**R**^**2**^		**beta**	***P *** **value**	**R**^**2**^
***hf*** **PWV**			.21	***cf*** **PWV**			.19
Age	.14	<.001		Age	.63	.062	
Systolic BP	.36	<.001		Systolic BP	.23	<.001	
BMI	.10	.009		BMI	.21	<.001	
Triglycerides	.07	.062		Triglycerides	.06	.075	
				Current smoking	.06	.085	
***fa*** **PWV**			.21	***ba*** **PWV**			.34
Systolic BP	.32	<.001		Age	.09	.003	
BMI	-.13	.001		Systolic BP	.42	<.001	
Heart rate	.07	.051		Heart rate	.09	.005	
Triglycerides	.15	<.001		Triglycerides	.16	<.001	
Current smoking	.09	.010		Current smoking	.06	.049	

*BP* blood pressure, *BMI* body mass index, *LDL-C* low density lipoprotein cholesterol, *PWV* pulse wave velocity, *hf* heart-femoral, *cf* carotid-femoral, *fa* femoral-ankle, *ba* brachial-ankle.

^a^The stepwise regression was performed for backward removal (p < .1) after adjusting for race as a lockterm. Each model includes age, systolic BP, heart rate, BMI, triglycerides, total cholesterol/HDL-C ratio, glucose, insulin, current smoking, and alcohol drinking as predictor variables. Triglycerides and fasting insulin were log-transformed.

Finally, based on R^2^ values in the multivariable adjusted models, the CV risk factors explained 21%, 19%, 21%, and 34% of the total variances in *hf*PWV, *cf*PWV, *fa*PWV, and *ba*PWV, respectively (Table 4).

## Discussion

Among healthy men aged 40 – 49, *cf*PWV correlated significantly and strongly with central *hf*PWV, whereas *ba*PWV correlated significantly and moderately with both central *hf*PWV and peripheral *fa*PWV. In the associations with CV risk factors, *cf*PWV shared common significant correlates with *hf*PWV and *fa*PWV, i.e., systolic BP and BMI. However, BMI was positively associated with *hf*PWV and *cf*PWV, and negatively associated with *fa*PWV. Meanwhile, *ba*PWV shared common significant correlates with *hf*PWV, i.e., age and systolic BP, and also with *fa*PWV, i.e., systolic BP, triglycerides, and current smoking. Thus, systolic BP was uniformly associated with all the regional PWVs. *cf*PWV was consistent with central *hf*PWV, while *ba*PWV was consistent with both central and peripheral PWVs in their associations with CV risk factors other than systolic BP.

We found that the peripheral arteries were stiffer than the aorta in our participants of men aged 40 – 49 based on the regional PWV values. Generally, such a difference is present in younger and middle-aged populations but not in older populations [25]. The Framingham Heart Study revealed that carotid-brachial PWV was greater than *cf*PWV in that segment of the aorta and the proximal central artery in the < 60 aged group, but *cf*PWV exceeded carotid-brachial PWV in the > 60 aged group of 521 men and women [16]. These results may be dependent on age-related decreases in the regional distribution of elastin contents found in the central elastic arteries. Studies have reported that the slope of the age-related increase in stiffness by arterial segments was greater in the aorta than in upper-limb or lower-limb arteries [25].

In our participants aged 40 – 49, *ba*PWV reflected mixed properties of the aortic and peripheral arteries, which can be primarily explained by the results of the correlation coefficients of *ba*PWV with each of *hf*PWV (r = .47), *cf*PWV (r = .42), and *fa*PWV (r = .62). Sugawara et al. [13] compared *ba*PWV with both *cf*PWV and *fa*PWV in 406 Japanese healthy adults aged 18 – 76 years, and reported correlation coefficients between *ba*PWV and *cf*PWV (r = .76) and between *ba*PWV and *fa*PWV (r = .76). Compared to the present study, the relatively greater magnitude of correlation between *ba*PWV and *cf*PWV may be due to a wider age range of their study population. In this context, *ba*PWV may reflect properties of aortic stiffness in a gradually increasing manner with aging. Moreover, *ba*PWV also reflects the properties of the proximal brachial arteries which are used to derive this measure. A few studies, including the Bogalusa Heart Study, reported that the proximal brachial arteries, similar to elastic carotid arteries, had an age-related decrease in distensibility, suggesting that they may behave differently from other muscular arteries (e.g., femoral or radial arteries) [14,26-28].

*ba*PWV was significantly associated with CV risk factors such as age, systolic BP, heart rate, triglycerides levels, and current smoking status, some of which were common factors with either *hf*PWV or *fa*PWV. Moreover, *ba*PWV was explained by these CV factors with relatively greater variance (R^2^ = .34) compared to *hf*PWV (R^2^ = .21), *cf*PWV (R^2^ = .19), and *fa*PWV (R^2^ = .21). Tsuchikura et al. [17] identified characteristics of *ba*PWV in comparison with *hf*PWV and *fa*PWV in associations with CV factors in 2,806 participants, including patients with diabetes mellitus, hypertension, or chronic kidney disease. *ba*PWV, *hf*PWV, and *fa*PWV shared the risk factors of higher age and the presence of diabetes and chronic kidney disease. Meanwhile, even though the mechanism explaining the predictive ability of *ba*PWV for the risk of cardiovascular diseases is still unclear, Vlachopoulos et al. [29] have demonstrated in a meta-analysis that an increase in *ba*PWV of 100cm/sec corresponded with an increase of 12%, 13%, and 6% in total cardiovascular events, cardiovascular mortality, and all-cause mortality, respectively. Therefore, the potential of *ba*PWV in the associations with CV risk should be further studied pathophysiologically and methodologically.

As expected, the association of age with arterial stiffness differed by arterial segments. Age was significantly associated with aortic stiffness (i.e., *hf*PWV), rather than peripheral and muscular arteries (i.e., *fa*PWV). As reported consistently in previous studies [7,15], the large elastic arteries near the heart stiffen with age by the reason of a decrease in elastin content. On the other hand, peripheral muscular arteries may be less or not affected by age because of their more vascular smooth muscle cells and less elastin, although there have been some controversies [7]. Unlike previous findings [14], our finding showed a lack of the significant association between *cf*PWV and age. It may be due to small variability in *cf*PWV within one-decade aged group (i.e., 40-49years).

Our results have shown that systolic BP was associated with both aortic stiffness as measured by *hf*PWV and peripheral stiffness as measured by *fa*PWV. Previously, Cecelja et al. [8] confirmed in a meta-analysis of 12 studies that BP was independently associated with *cf*PWV, and the contribution of standard CV risk factors other than age and BP were small or nonsignificant in multivariable regression models. Meanwhile, our data showed that the magnitude of the association between systolic BP and *fa*PWV (beta = .32) was similar to that between systolic BP and *hf*PWV (beta = .36), which paralleled the findings of previous studies [30,31].

In our study, triglycerides and smoking were significantly and positively associated with *fa*PWV, but not with *hf*PWV. Previously, no studies have reported such associations of CV risk factors with peripheral (leg) stiffness compared to aortic stiffness in a healthy population. Kimoto et al. [18] reported associations of CV risk factors with *hf*PWV vs. *fa*PWV among 161 diabetes patients and 129 healthy subjects. As a result, age and BP were significantly associated with both *hf*PWV and *fa*PWV, whereas the presence of type II diabetes was significantly associated only with *hf*PWV [18]. In addition, the relation of the stiffness of the peripheral (leg) artery to cardiovascular outcomes has been found to be nonsignificant in patients with certain diseases [30,32]. Tillin et al. [30] reported no significant associations of *fa*PWV with coronary calcification and carotid intima-media thickness in patients with coronary artery disease, in contrast to significant associations of *cf*PWV. Thus, compared to aortic PWV, the clinical importance of the peripheral PWV remains to be still established.

We found that there was an inverse association between BMI and *fa*PWV. In several cross-sectional studies, increased BMI has been reported to be positively associated with the aortic stiffness measured by *cf*PWV [5,33,34], which parallels with the positive associations of BMI with each of *hf*PWV and *cf*PWV in our present study. However, no previous studies reported the association between BMI and peripheral stiffness. A few studies have reported a negative association between *ba*PWV and BMI. Tomiyama et al. [10] reported that *ba*PWV was significantly and negatively associated with BMI among 7,881 healthy Japanese men, but not among the women. Physiologically, arterial stiffness may be related to the distensibility and arterial diameter of the artery. Zebekakis et al. [33] reported that BMI was related to increased diameter and decreased distensibility in femoral arteries, and this increased diameter associated with BMI was significantly greater in middle-aged men (age 40) than in older aged men (age 60). Weight gain may be related to increased regional blood flow to adipose and non-adipose tissues [35]. Therefore, the inverse association between peripheral PWVs and BMI may be attributable to the effort of increasing the diameters of peripheral arteries, a compensating mechanism to minimize decreased distensibility in middle-aged men.

The strengths of the study include the fact that this is the first study to explore differential characteristics of *cf*PWV and *ba*PWV by simultaneously comparing these with the central and peripheral PWVs in a healthy middle-aged group. The limitations of the study include the cross-sectional nature of the study design, which cannot distinguish between cause and effect, especially between BP and PWV. The limited age range (i.e., 40–49 years) and only male population may lead to lack of generalizability for our findings to populations of wider age ranges and to female populations. In addition, we used the formula for calculating the path lengths for *hf*PWV, *fa*PWV and *ba*PWV, which was derived from the body sizes of the Japanese population. This formula may not be always congruent with body sizes of the White American or African American male population groups.

## Conclusions

Among healthy men aged 40 – 49 years, *cf*PWV correlated strongly with central PWV, and *ba*PWV correlated with both central and peripheral PWVs. In the associations with CV risk factors, *cf*PWV was consistent with central PWV, while *ba*PWV was consistent with both central and peripheral PWVs. Therefore, *cf*PWV mostly reflected properties of central arterial stiffness, while *ba*PWV reflected mixed properties of both central and peripheral arterial stiffness.

## Competing interests

The authors declare that they have no competing interests.

## Authors’ contributions

The authors’ contributions were as follows – JC and AS developed the hypothesis of this study and prepared the manuscript draft; JC, AS, CS, EBM, KM, BJW, and TBS involved in data collection; JC led the data analyses; AS, HU, SL, KM, LV, RHM, RWE, KST, and LHK provided expert consultation on data interpretation. All authors were involved in the review and revision of the manuscript and gave final approval of the version to be published.

## Pre-publication history

The pre-publication history for this paper can be accessed here:

http://www.biomedcentral.com/1471-2261/14/5/prepub
